# Managing and mitigating future public health risks: Planetary boundaries, global catastrophic risk, and inclusive wealth

**DOI:** 10.1111/risa.17703

**Published:** 2025-01-18

**Authors:** Eoin McLaughlin, Matthias Beck

**Affiliations:** ^1^ Edinburgh Business School Heriot‐Watt University Edinburgh UK; ^2^ Health application Lab (HeAL) Heriot‐Watt University Edinburgh UK; ^3^ Cork University Business School University College Cork Cork Ireland

**Keywords:** global catastrophic risk, planetary boundaries, wealth

## Abstract

There are two separate conceptualizations for assessing existential risks: Planetary Boundaries (PBs) and global catastrophic risks (GCRs). While these concepts are similar in principle, their underpinning literatures tend not to engage with each other. Research related to these concepts has tended to be siloed in terms of the study of specific threats and also in terms of how these are assumed to materialize; PBs attribute global catastrophes to slow‐moving and potentially irreversible global changes, while GCRs focuses on cataclysmic short‐term events. We argue that there is a need for a more unified approach to managing global long‐term risks, which recognizes the complex and confounded nature of the interactions between PBs and GCRs. We highlight where the PB and GCR concepts overlap and outline these complexities using an example of public health, namely, pandemics and food insecurity. We also present an existing indicator that we argue can be used for monitoring and managing risk. We argue for greater emphasis on national and global ‘‘inclusive wealth’’ as a way to measure economic activity and thus to monitor and mitigate the unintended consequences of economic activity. In sum, we call for a holistic approach to stewardship aimed at preserving the integrity of natural capital in the face of a broad range of global risks and their respective regional or global manifestations.

## 1 INTRODUCTION

Five known mass extinction events occurred on this planet in the last 3.5 billion years, and it is argued that we are currently living through a sixth (Barnosky et al., 2011). There have been additional extinction events identified that are believed to be associated with extraterrestrial (i.e., meteors, asteroids, and comets) events (Raup & Sepkoski, 1983). While humanity as a species has not been exposed to such an extinction event, recent evidence suggests our ancestors may have been endangered and survived an unknown catastrophic event 117,000 years ago (Hu et al., 2023). As humanity has progressed, it has become the first species that is able to remake its world, but also has the capacity to destroy it too (Jha, 2011).

**FIGURE 1:**
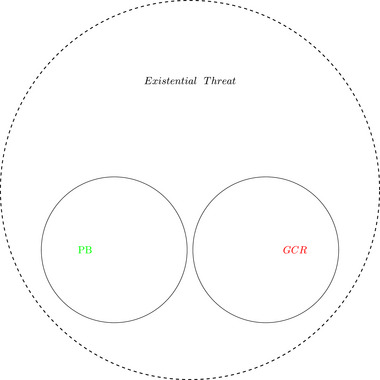
Two conceptualizations of existential threats.

Recent global concerns, such as the climate crisis, Covid‐19 pandemic, wildfires, and the reemergence of the risk of nuclear war, have created a heightened awareness of global catastrophic risk (GCR) (although perhaps not the expression itself) among the public and the mainstream media.^1^ Yet, such GCRs have traditionally been overlooked in national risk assessments and Boyd and Wilson (2023) see this as a shortcoming of such exercises. Boyd and Wilson (2020) recently highlighted the ‘‘small but growing’’ subfield of risk research that focuses on GCR. Another underutilized field of risk research, planetary boundaries (PBs), shares many similarities with the GCR fields approaches to monitoring and evaluating existential risks to humanity (although the two approaches to existential risk remain siloed [see Figure 1 for a possible conceptualization of the relationship between the two approaches]). Here, we highlight the areas of overlap between the two approaches and how an holistic approach to risk monitoring and management may be beneficial by applying the concepts to a particular case of public health, namely, pandemics. Further, we answer Boyd and Wilson's (2020) call for greater efforts at mitigating GCRs by highlighting an off the shelf concept and metric, the measurement of so‐called ‘‘inclusive wealth’’ (*IW*), that we believe can be repurposed as a means to mitigate certain existential risks.

The contribution of our study is that we critically evaluate both PB and GCR approaches, we also incorporate an additional element for monitoring risk (*IW*). The existing literature has been siloed and we are effectively bringing together three distinct strands of literature. We critically review the literature on PBs, GCRs, and *IW*. We stress the emphasis on a critical review because the PB and GCR literatures are old concepts but the interaction between them is poorly recognized and rarely explicitly highlighted.

The following sections of our article first establish a risk typology, and then summarize some key ideas in the PB and GCR domain and expand on the idea of risk interaction. We then discuss emerging research on the link between PBs and some GCRs, namely, through food security and pandemics. We finish by highlighting the importance of monitoring *IW* as a means for global and national risk management.

## 2 ESTABLISHING A RISK TYPOLOGY

PBs are parameters beyond which there is a level of risk that can undermine the long‐term survival of humanity and act as markers of “safe operating spaces” for humanity that can be quantified. It is posited that once these boundaries are crossed, the consequences for human survival can be irreversible (Richardson et al., 2023; Rockström et al., 2023; Steffen et al., 2015). GCRs also assess risks to humanity that have potential for enormous harm at a global scale, but the GCR literature differs from the PB literature in that it contemplates a broader array of global risks to the survival of humanity, such as artificial intelligence, biothreats and pandemics, and climate change (Bostrom & Ćirković, 2008; Ord, 2020).

While the PB and GCR concepts themselves are distinct, there is an element of overlap between the two conceptualizations of existential risk.^2^ The linkage between PB and GCR can be traced back to Beck (1992, 2009), who focuses on risks that are the by‐products of modernization/industrialization, such as climate change and nuclear waste. Take the example of climate change that is both a specific PB and is also categorized as a GCR. More recently, Baum and Handoh (2014) have called for dialog between PB and GCR researchers to facilitate studies of the human consequences of global environmental change. They assume human resilience to crossing a PB, but state that interactions with flawed socioeconomic institutions (e.g., a major supply chain failure) can create disastrous outcomes. While sympathetic of this view, we argue that the PB and GCR distinction should be carefully assessed and that the siloing of thought hampers the conceptual understanding of key global risk constellations beyond the confines of particular lens of risk. Our contribution is a critical clarification regarding the relationship between existing frameworks.

**FIGURE 2:**
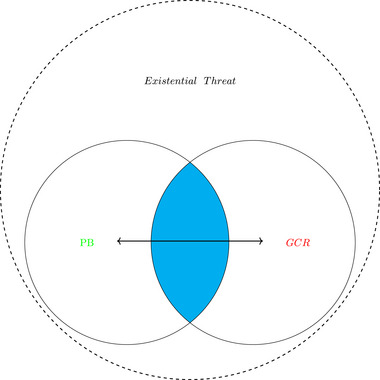
Speculative reorganizing conceptualizations of existential threats. *Note*. The line represents interactions between PBs and GCRs.

In the case of Baum and Handoh (2014), their specific focus was on global phosphorus biogeochemical cycle and how the PB approach could gain insights from the GCR research. Our application differs on two fronts: we focus on public health, pandemics in particular, and our aim is to highlight the overlap between the two conceptualizations of existential risk and the difficulty of attributing such risks to either paradigm. To fully appreciate the interacting network of current key global health risks requires us to consider most PBs as also having GCR characteristics and vice versa (although this applies to a subset of GCRs, see Table 1); we contend that the distinction between PB and GCRs is an obstacle to a more holistic understanding of a certain risk constellation. Put another way, rather than assuming that PB resilience is reduced by interactions between PB impacts and ‘‘faulty’’ socioeconomic institutions, should we not assume, as a matter of caution, that socioeconomic institutions are likely to be flawed in managing PBs, particularly as the complexity of these tasks increases, and that flawed responses, in turn, could quickly lead to catastrophic outcomes. This is now the direction in which some PB literature is headed; for example, Kemp et al. (2022) include researchers from both PB and GCR traditions. In essence, we argue that the siloing of PB and GCR research impedes monitoring and mitigation of risk in much the same way that siloing of human and animal health jurisdictions impedes responses to the containment of zoonotic diseases (Jerolmack, 2013). In essence, our argument is to reorganize these concepts and nest PBs within GCRs (see Figure 2).

**TABLE 1:** Global catastrophic risk by category and relationship to PB.

Categories	Risk	Directly related to PB?
Risks from Nature	Super volcanoes	Y
	Meteors, asteroids, and comets	Y
	Supernovae and solar flares	Y
	Agricultural failure such as a plant virus or superweed	Maybe (if this is consequence of crossing a PB)
	Extreme Ice Age	Maybe (if this is a consequence of crossing PBs)
	Rogue Black hole	N
	Anoxic event from oxygen depletion	Maybe (if this is a consequence of crossing PBs)^a^
Risk from unintended consequences of human activity	Climate change	Y
	Pandemics	Y (Biodiversity boundary)/N (if bioengineered)
	Unaligned Artificial Intelligence	N
	Social collapse: Political intervention in reproduction; Colony collapse disorder Voluntary extinction	Y (if PBs exceeded)
	Accident in high‐energy physics	N
	Ecosystem collapse	Y
	Infertility due to chemicals or biology	Y (if due to chemicals)
	Unable to procreate due to genetic engineering	N
Risk from hostile acts	Nuclear War	Y
	Bioterrorism	Y
	Nanotechnology	N

*Note*: Risk classification derived from Bostrom and Ćirković (2008) and Tonn and Stiefel (2013). ^a^Anoxic events have been associated with periods of global warming (Meyer & Kump, 2008), which could be related to a PB or could be a natural phenomenon.

The argument for a holistic approach to existential risk is supported by the fact that there is no absolute demarcation line between PB and GCR risks. Bostrom and Ćirković (2008) offer some guidance on what would and would not constitute a GCR by defining damage, global in scale, in terms of deaths and economic damage. For deaths, the range for a catastrophe is between 10 thousand and 10 million (or more) fatalities, and for economic damages, it is $10 billion and $10 trillion (or more) worth of economic loss, ‘‘even if some region of the world escaped unscathed.’’^3^ This is similar in many respects to the definition of harm from crossing a PB threshold as outlined in Rockström et al. (2023), namely, ‘‘widespread severe existential or irreversible negative impacts on countries, communities and individuals from Earth system change, such as loss of lives, livelihoods or incomes; displacement; loss of food, water or nutritional security; and chronic disease, injury or malnutrition.’’

A recent example helps to demonstrate our argument: was Covid‐19 a PB or a GCR? With the Covid‐19 pandemic, researchers and international bodies have speculated about links between coronaviruses and human encroachment into wild habitats, or the degradation of ecosystems (Vidal, 2020), leading to calls for greater protection of biodiversity (e.g., WWF, 2020). These concerns have been framed within the PB or GCR paradigms (e.g., GCF, 2022; Talukder et al., 2022). Global excess deaths for Covid‐19 were estimated at 18.2 million from January 1, 2020 to December 31, 2021 (Wang et al., 2022), and arguably, this figure could have been higher were it not for the various mitigation efforts implemented globally. The economic costs associated with Covid‐19 were staggering; estimated costs for the United States alone amount to $16 trillion (Cutler & Summers, 2020). Under the definitions of Bostrom and Ćirković (2008) and Rockström et al. (2023), it is clear that Covid‐19 constitutes a GCR as pandemics are part of this framework, but also the PB framework as biodiversity pressures are a key PB. Yet, one could still question whether Covid‐19 falls under either a PB or a GCR framework, or both.

Once we can agree on a risk typology, the question becomes how these risks can be effectively mitigated and managed. Many GCRs are considered “natural risks” (e.g., see Table 1) and require geological and astronomical monitoring. Other GCRs—those driven unintentionally and intentionally by human actions—should also be monitored. The latter relate to nuclear or biological warfare, while the former can be the unintended consequences of economic activity. Effectively, this breaks risks into exogenous, risks that arise outside of human activities, and endogenous, risks generated by human activities. This brings us to the key question: how should we measure the economy to provide a better indicator of the impact of economic activity on the environment (e.g., on PBs)?

The conventional way to measure the economy is by calculating a nation's gross domestic product (GDP), which is a measure of an economy's income, and the change in GDP is used to measure economic growth (Stiglitz et al., 2009). GDP as a measure is a flow; it tells us what economic activity was over a certain time period, but it does not tell us what is happening to underlying stocks (the accumulated value of stocks at a point in time) that went into generating the flow (Hamilton & Hepburn, 2014). Stiglitz (2020) places particular emphasis on how there appears to be little correlation between GDP (levels and growth) and the mortality impact of Covid‐19.^4^ Therefore, instead of measuring the economy in terms of gross income, Dasgupta and Levin (2023), among others,^5^ argue that we should measure the *IW* of an economy whereby *IW* is composed of different capital stocks; physical, human, and natural capital (Dasgupta, 2021; Dasgupta & Levin, 2023). This, in turn, relates to wider calls for how we measure the economy (e.g., Nature, 2023).

**FIGURE 3:**
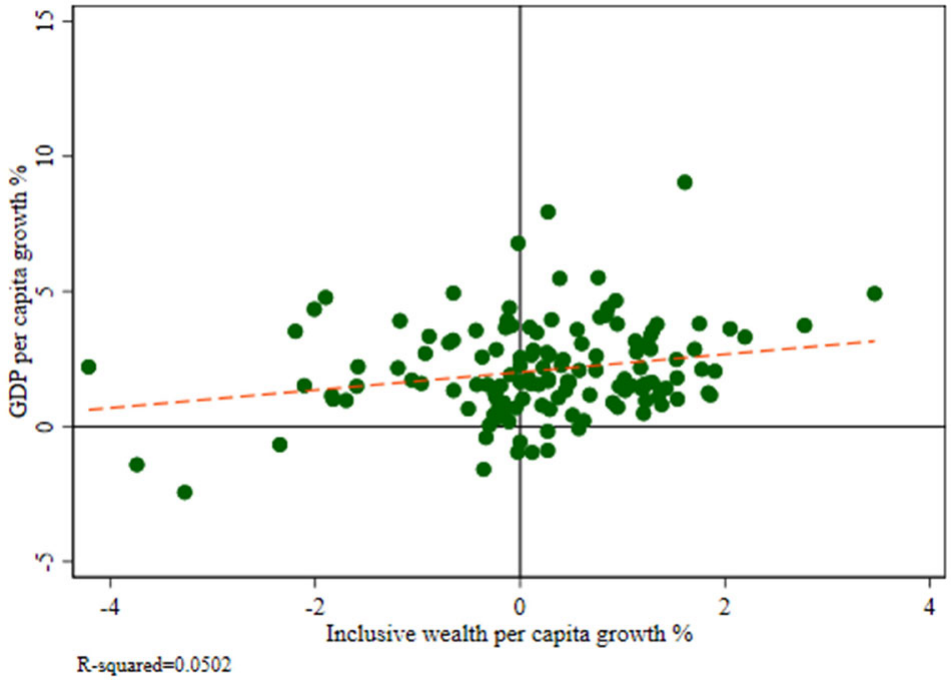
Scatterplot of GDP per capita growth and inclusive wealth per capita (1990–2014). *Sources*: UNEP (2018) & UN (2022). *Note*. Each point represents a country. There are 137 countries represented and the data are from the period 1990–2014.

*IW* sits at the core of our analysis. Our proposal for improved risk management centers on adopting a more careful and considered approach to maintaining the integrity of natural capital, broadly defined. Figure 3 highlights a recent empirical application of so‐called wealth accounting by United Nations Environmental Programme (2018).^6^ *IW* tracks changes in a country's stock of assets (manufactured, natural, and human). Figure 3 shows that the growth in *IW* per capita differs significantly from the growth in GDP per capita (UNEP, 2018; Yamaguchi et al., 2019). Over the sample period (1990–2014), 23 countries showed negative growth in GDP per capita, while 51 countries experienced negative growth in their *IW* per capita. Thus, we would argue for greater focus on *IW* as an indicator for monitoring what is happening to our natural capital, while using GDP growth for monitoring short‐term fluctuations (Dasgupta & Levin, 2023). This would help provide an indicator that can monitor risks that are endogenous to our economic system. While the Dasgupta (2021) review on biodiversity uses the *IW* framework in the context of PBs only, the incorporation of *IW* here is novel as the focus is on both PBs and GCRs.

**FIGURE 4:**
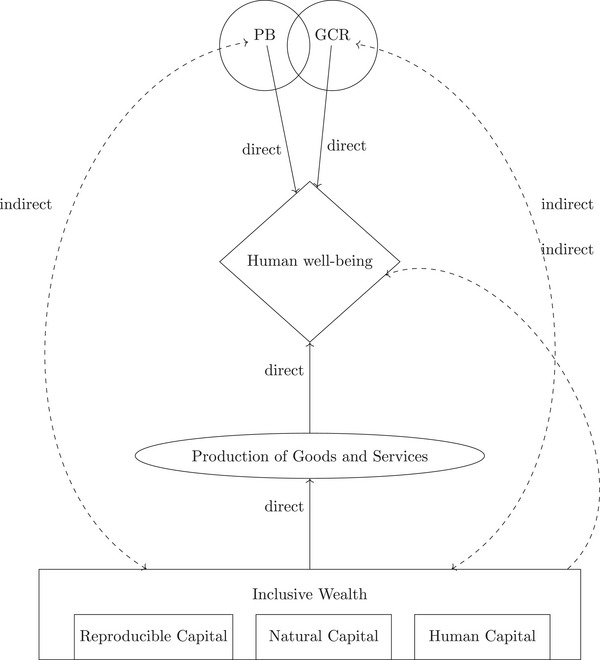
Conceptualization of link between PB/GCR and IW. *Note*. Inclusive Wealth Schema follows Barbier (2015) with our interpretation of PB‐GCR overlaid on this.

The conceptual link between the *IW* approach and the GCR‐PB framework comes from their focus on human well‐being. For example, Rockström et al. (2023) and Baum and Handoh (2014) motivate their analysis in terms of human well‐being, this is also central to *IW* (e.g., Arrow et al., 2012; Polasky et al., 2019; Dasgupta & Levin, 2023)). From this perspective, what Covid‐19 has highlighted is the importance of our *IW*, in particular, our natural capital (Dasgupta, 2021); where natural capital is usually defined as all “gifts of nature”—renewable and nonrenewable resources, such as coal and forests; ecosystems that generate a flow of services over time; and the global climate system (Hanley et al., 2015). *IW* is typically not discussed in the PB or GCR literatures. Wealth (note, not *IW*) is discussed by Beck (1992), although flows and stocks appear to be conflated that ultimately damages the applicability of the social risk concepts to the analysis of environmental risks. We have illustrated this linkage in Figure 4.^7^

Covid‐19 highlighted how the global economy is vulnerable to GCR‐PBs—in terms of PBs the biodiversity boundary and for GCRs, it is pandemic—but that the GCR‐PBs risks driven by economic activity are underestimated because the most widely used metrics for measuring economic activity tell us little of what is happening to the underlying natural capital (Coyle, 2017). This *IW* measure is therefore an approach that we would advocate for risk management by placing greater emphasis on the management of *IW*, both nationally and globally. Thus, while Boyd and Wilson (2020) have criticized international bodies, such as the UN, for a lack of engagement with catastrophic risk, some aspects of their preexisting body of work can be repurposed as a tool to mitigate certain aspects of PBs and GCRs. In particular, monitoring *IW* can warn policymakers when economic activity is undermining natural capital.

## 3 PLANETARY BOUNDARIES

PBs are one accepted way of thinking about existential global risks. Works based on the PB framework seek to describe estimated levels of environmental change that minimize risks to the survival of humanity, while humanity's role in driving change in earth systems is explicitly taken into account (Richardson et al., 2023; Rockström et al., 2009a, 2009b; Steffen et al., 2018). PBs‐based research seeks to set boundaries that represent quantifiable markers of ‘‘safe operating spaces’’ for humanity that, once crossed, could have irreversible consequences for human survival. Climate change and biosphere integrity are ‘‘core’’ boundaries that interact with other boundaries (Steffen et al., 2015, 2018); with recent updates focusing on nuancing existing boundaries (Wang‐Erlandsson et al., 2022), incorporating novel boundaries (Persson et al., 2022), or discussing governance for the boundaries (Rockström et al., 2024). However, since the 2015 iteration, there is a now a clear hierarchy to PBs with climate change now sat at the apex.

The PB approach distinguishes boundaries that are buffer zones of uncertainty before a critical threshold, while these thresholds are defined as zones once crossed could push the climate out of the hospitable Holocene conditions in which life has thrived on Earth. This is perhaps best explained in Rockström et al. (2023) who describe the thresholds as ‘‘tipping points that irreversibly destabilize the Earth system’’ and the safe boundaries are determined by ‘‘assessments of tipping point risks among local and regional tipping elements, evidence on declines in Earth system functions, analyses of historical variability and expert judgement.’’ The same researchers argue that there is an increased likelihood of tipping points in the earth system and that there could be a cascade of tipping points (Lenton et al., 2019).

In a recent iteration of the PBs, Rockström et al. (2023) refer to Earth system boundaries (ESBs). These are distinct from PBs in that they focus on different scales of analysis. At a global level, ESBs are identical to PBs, but at subglobal levels, ESBs do not equate to PBs. This means that at a local level, ESBs are context specific and do not constitute a global risk. The boundaries are conservative estimates, reflecting uncertainty surrounding the probable impact of a risk while incorporating normative, or rather ‘‘subjective,’’ judgments about risk by adopting the ‘‘precautionary principle’’ (Rockström et al., 2023). Biermann and Kim (2020) are critical of the boundaries as a normative exercise with no input from civil society or government in the selection of the boundaries; the decision over what constitute boundaries were left to a narrow group of technocrats. In the original PB study, of the 29 authors listed, none were from the global south a feature that hampered the adoption of the framework—effectively reopening debates surrounding development and environment that have plagued North/South tensions since the 1970s (Saunders, 2015).

According to Rockström et al. (2009a), there are nine boundaries classified, and these are outlined in Table A1. While the boundaries have remained the same, some of the indicators used to quantify the boundaries have changed since the original study and the most recent iteration (Richardson et al., 2023). While the individual boundaries are specified, the interlinkages and interaction between the various processes effectively make them a composite index of anthropocentric‐driven risk to the Earth System and feedbacks within the Earth System are continually emphasized as a risk within the PB literature (Steffen et al., 2007). Although not explicitly examined in the original studies (Rockström et al., 2009a, 2009b), anthropocentric climate change is now often given a first‐order ranking, with the ‘‘2°C guardrail’’ set as a boundary.^8^ In subsequent work, climate change and biosphere integrity are classified as ‘‘core’’ boundaries that interact with other boundaries (Richardson et al., 2023; Steffen et al., 2015). Feedback processes often depend strongly on climate change (Steffen et al., 2018).

Rockström et al. (2023) advocate the use of a so‐called ‘‘3I justice criteria’’ that involves a trifecta of justice; interspecies, intergenerational, and intragenerational justice. In practice, this means making ESB boundaries tighter than they were under the older PB framework but the thresholds themselves are unchanged. However, this incorporation of interspecies justice has not led to an approach beyond minimizing human harm. An example of this new focus relates to the climate change boundary, a more stringent ‘‘≤1.5°C guardrail’’ is chosen instead of the previous ‘‘2°C guardrail,’’ this boundary is chosen to ensure a more just social outcome (Rockström et al., 2023), but the underlying threshold/tipping point is unchanged. In an update to the monitoring of PBs, Richardson et al. (2023), while not adhering to the ‘‘3I justice criteria’’ of Rockström et al. (2023), also place emphasis on ‘‘safe’’ levels for each boundary based on preindustrial levels. There is also an upper level derived for each boundary, and based on this framework, there are now six processes that are currently beyond their boundary, and climate change and biodiversity loss are dramatic examples of these.

Many studies rely on PBs as motivating factors (Downing et al., 2019) and derive their analysis of future threats to public health from the perceived breach of PBs (McMichael, 2013). Most notable is the Rockefeller‐Lancet Commission on planetary health that draws on all PBs to develop measures of human and planetary health (Whitmee et al., 2015). A somewhat different version of ‘‘safe operating spaces’’ for our health has been developed by Dasgupta (2020, 2021) that specifically focuses on one boundary, biodiversity, arguing that humanity should live within the safe operating space of the biosphere to prevent spillovers of infectious disease from vulnerable wildlife populations.

One critique of the PB literature centers on its anthropocentric^9^ focus, which restricts analysis to human influence on environments instead of viewing these interactions as two‐way process, where environments can influence human activity. This has led to calls to incorporate socioenvironmental interactions within PBs (Donges et al., 2017). A recent PB study responds by examining interactions between various PBs (Lade et al., 2020), and this is continued in Richardson et al. (2023) who see these as systemic risks (see discussion in Section 5). These interactions focus on how human activity can impinge on the PBs, rather than stating how PBs affect human activity. Thus, while illustrating an extensive list of interactions, Lade et al. (2020) have left space for further research in this area. Interactions have, however, long been central to other work in this tradition. For example, Meadows et al. (1972) thought that there were interactions between environmental and economic activity that could have regional and global consequences. Similarly, Ord (2020)’s work on climate change saw greater risk through indirect (interaction) effects as compared to direct effects. More recent work in the PB tradition has focused on catastrophic climate change and discusses how climate change could interact with other PB thresholds and other threats such as inequality and fragile states.^10^ For example, Kemp et al. (2022) argue that climate change could reinforce other interacting threats and highlight that there is an overlap between areas with projected extreme heat in the future (c. 2070) and what are classified as vulnerable states today.

## 4 GLOBAL CATASTROPHIC RISKS

**FIGURE 5:**
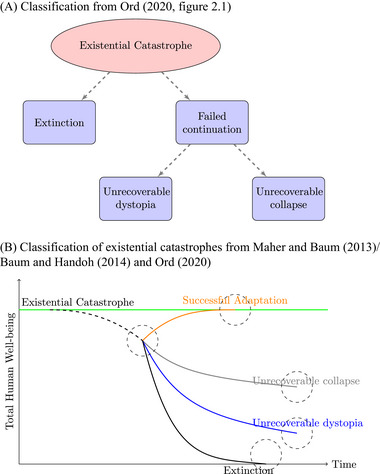
Classification of existential catastrophes. *Note*. The horizontal line represents baseline well‐being of humanity.

The central focus of the GCR literature is on risks to humanity defined by their scope, intensity, and probability (Bostrom, 2002; Rees, 2003, 2018; Ord, 2020).^11^ The scale is global, not local or regional, and the affected group is all of humanity not specific groups. In this framework, a multitude of independent small events does not constitute an existential catastrophe. For an event to be catastrophic requires a single decisive event, or simultaneous events (Baum et al., 2013), or a singular chain of events (Tonn & MacGregor, 2009), with permanent, irreversible outcomes. Outcomes then range from extinction to failed continuation of the remainder of humanity (Ord, 2020)—see Figure 5 (Panel a). Maher and Baum (2013) approach the classification in a similar way, presenting the classification over time in terms of anthropocentric values (human well‐being), although they also include the possibility for adaptation to the catastrophic event. Figure 5, Panel b modifies the Ord (2020) classification tree to fit with Maher and Baum (2013).^12^

The range of possible scenarios is great and ascertaining the likelihood of such events is difficult, given that the lack or absence of observational data (Helbing, 2013; Kuhlemann, 2019; Tonn & Stiefel, 2013). As we noted in the introduction, there are five known extinction events. There was only one near extinction event for humanity identified.^13^ Hu et al. (2023) estimate that about 98.7% of human ancestors were lost and that the human population fell to a level of 1280 breeding individuals during the Pleistocene transition; this low population level lasted for 117,000 years. However, an exact understanding of the ultimate drivers of this event are unclear and it is attributed to climactic changes.

If there is a distinguishing characteristic of GCR, it is that it focuses on specific low probability but high impact events. Bostrom and Ćirković (2008) identify three key categories of GCRs, risks from nature, risks from unintended consequences of human activity, and risks from hostile acts. Similarly, Tonn and Stiefel (2013) classify risks by source sociopolitical, technological, chemical/nuclear, and biological and these are further elaborated in Table 1.

Table 1 highlights whether PBs are directly related to the various GCRs outlined. While some of this categorization is speculative, other areas show a clear association. For example, climate change is clearly related to both concepts, while others may not at first glance appear to be so. However, it could be argued that some natural hazards (e.g., super volcanoes, giant meteors, etc.) would have an effect on specific PBs (e.g., reduced levels of oxygen, light, food, etc., that could threaten human subsistence). For example, climate variability is naturally driven by volcanic eruptions. This was most spectacularly evident with a series of volcanic eruptions in the 500s (Sigl et al., 2015) that led to global famines (following an eruption in 536 AD—Newfield, 2018) and pandemic (the Justinianic Plague [Mordechai et al., 2019] that followed another major eruption in 539/540).^14^ A similar line of reasoning would relate the risks from hostile acts to impacts on PBs. However, there is no absolute demarcation line between risks relevant to PB and GCR. Recent research suggested that GCR events significantly disrupt a critical system (Avin et al., 2018), which may or may not be part of a PB; but this does not create a clear distinction. One of the traditional distinctions between PBs and GCRs are the assumed spread and predictability of the risks concerned: With PBs, as defined above, being gradual and predictable and GCRs being sudden and relatively unpredictable. Or, in the view of Richardson et al. (2023), that PBs should be viewed as a system, whereas GCRs can be independent, simultaneous, or chain events. One obvious problem with this distinction it that there are no clear rules for defining events as sudden versus predictable, with much of this depending not only on detailed knowledge of specific events but also specific, often highly politicized, interpretive frames.

A simple example of this would be the question whether we could consider the 2014–2016 Ebola outbreak in West Africa, the largest and most complex of its kind (WHO, 2021a) as predictor of the Covid‐19 pandemic, or alternatively whether the SARS pandemic of 2003 should be considered as such. Regarding the latter, Kleinman and Watson's (2005) edited book posed the question whether SARS was a prelude to the next pandemic. In hindsight, it appears to have similarities in terms of its coronavirus origins and the importance of dispersion (“superspreaders”) in transmission (Royal Society, 2020; Sun et al., 2021). However, when Edwin Kilbourne (2006), one of the most respected virologists of the 20th century and a contributor to the Bostrom and Ćirković (2008) edited volume on GCRs, reviewed this work in *JAMA*, he dismissed the question with the pithy line ‘‘8000 cases do not a pandemic make.’’ Obviously, Kilbourne's comment has limitations in light of modern experience, and this definition of a pandemic also differs from the classical definition of a pandemic as ‘‘an epidemic occurring worldwide, or a very wide area, crossing international boundaries and usually affecting a large number of people’’ (Kelly, 2011). Clearly, much depends on which facts we focus on, as well as how we define a surprise event as compared to a predictable chain of events. Notwithstanding, the strong intellectual foundation of the ‘‘Black Swan’’ concept in terms of degenerate meta probabilities (Taleb, 2007), fat tails (i.e., rare, but high impact, events), or extreme value events (Hochrainer‐Stigler, 2020), there too remains a subjective element to the nature of acknowledged surprises, in terms of the fact that the recognition of the fragility of the earth as system is going to make us more likely to expect risks to materialize and interact with each other.

As noted above, Baum and Handoh (2014) call for an integration of PBs and GCRs paradigms, by integrating aspects of each paradigm to create a what they labeled Boundary Risk for Humanity and Nature (BRIHN). However, given the wide array of GCRs (see Table 1), it is unclear how this conceptual framework is operationalized, and we feel that this categorization is unproductive. The separation between PB approaches and GCRs importantly provides an insufficient theoretical basis for conceptualizing increasingly relevant interactions such as those between climate change and health risks. Helbing (2013) emphasizes that the role feedback loops can play in exacerbating risks and shocks as they are cascading and creating CGRs. The link between climate change and the incidence of vector‐borne disease^15^ such as malaria and tick‐borne diseases is well established (Bouchard et al., 2019; Campbell‐Lendrum et al., 2015; ICS, 2017; McMichael et al., 2003; Shahhosseini et al., 2021). Also, pathogens are known to adapt to increases in temperature, creating drug‐resistant fungal species that have emerged in the past decade (Casadevall et al., 2019). Health impacts of climate change feature in climate economy models (Nordhaus, 2017) and are deemed to be ‘‘one of the major concerns’’ of public health (Romanello et al., 2022). The focus in these models is often on the spread of tropical and subtropical diseases (such as malaria, dengue, and yellow fever),^16^ as well as interaction of air and water pollution with both higher temperatures and more frequent flooding (Nordhaus & Boyer, 2000). But it is likely that such discussions underestimate risk if there are significant risk interactions. The *Dasgupta Review of the Economics of Biodiversity* accentuates the link between pressures on biodiversity, in itself a PB, and health risks such as Covid‐19. By this the *Report* warned about the likelihood of greater zoonotic spillover if land use change, one of the main drivers of biodiversity loss, continued to encroach on the habitats of wild animals, a reservoir of zoonotic disease (Gottdenker et al., 2014).^17^ The *Review* argues that a greater focus on a holistic view of assets would have given policymakers warnings about possible risks (Dasgupta, 2021).

Baum and Handoh (2014) cogently propose that humans could be resilient to crossing a PB, but if there is an interaction with socioeconomic institutions (e.g., a supply chain failure in relation to life‐saving drugs), this might create a disastrous outcome, which is a scenario to which we now need to add the possibility of a formerly life‐saving drug (e.g., due to antimicrobial resistance) losing its potency while the development of an alternative consumes time and material resources. While the former case of a supply chain failure may not be a GCR, rather a more general risk, the latter, antimicrobial resistance, would constitute a GCR given the global reach of the problem (Murray et al., 2022). Both scenarios undermine the rationale for separating the two approaches as this is largely based on the flawed idea that the latter can be adequately mitigated while the former cannot.

One conventional GCR‐type argument is that pandemics, as GCRs, can be mitigated and/or eliminated via public health interventions (e.g., Kilbourne, 2008). The view that pandemics can be easily mitigated is highly problematic on a number of counts. First, WHO research indicates that many diseases resulted in pandemics that have not been eliminated but rather are prone to ‘‘flare ups,’’ at times of pandemic proportions. Where ‘‘flare ups’’ occur, public health responses are sometimes insufficient with, for example, unequal access to vaccines; an issue that became evident during the 2009 influenza pandemic (Jorgensen et al., 2013). Second, increased presence, and or detection, of zoonotic pandemics again indicate a blurring of boundaries where climate related risk, pressures on food security, and population pressures make traditional PB and GCR boundaries increasingly difficult to define. This is exemplified by the recent avian influenza outbreak in the Russian Federation that, albeit identified as low risk, calamitously paralleled the ongoing Covid‐19 pandemic (WHO, 2021b).

## 5 INTERACTION OF PLANETARY BOUNDARIES AND GLOBAL CATASTROPHIC RISKS

**FIGURE 6:**
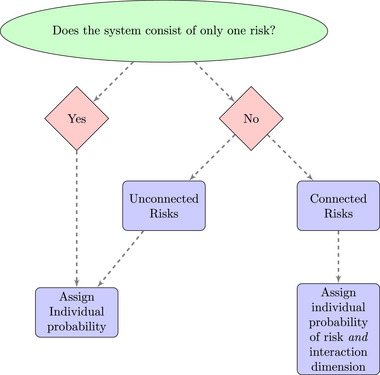
Risk within a complex system. *Note*. Figure based on description of systematic risk and dependencies from Hochrainer–Stigler (2020).

Interactions of risky and near‐catastrophic events create risks at several levels (Helbing, 2013).^18^ There is discussion within the GCR literature on the interaction of risks, with research focusing on the impact on food security of a GCR, such as a pandemic or nuclear war (Helfand, 2013; Huff et al., 2015), but this seems less clearly discussed in the PB literature.^19^ Continuous interactions, at different scales, meanwhile, are seen as integral to the function of social–ecological systems (Reyes et al., 2018). Interactions are foundational to the understanding of complex systems and the aggregation of such interactions can lead to properties greater than the individual components (Cillers, 2013; Jensen, 2023), with key properties of complexity relating to interactions.^20^ Figure 6 is a representation of the approach taken in systematic risk, if there is only one risk within a system, then an assigning probability of risk to that element will suffice, but if there are multiple elements at risk, then the question becomes whether or not they are related and then, if they are related, what is this relationship (Hochrainer‐Stigler, 2020). This relationship may imply dependence (interpreted as correlation) of risks or “tail dependence,” whereby the relationship is found in the tails of the distribution. Thus, an important consideration is whether the interaction of risks implies subadditivity, additivity, or supraadditivity (synergistic interaction) of risk; evidence suggests that the interaction of risks can amplify risks (e.g., Arrigo et al 2020). The challenge with such complex interactions is the difficulty in preparing, controlling, and managing these interactions once they do occur (Helbing, 2013). Synergistic risks would be of greatest concern in the context of PBs and GCRs as the impact would be greater than that of a risk in isolation.

An historical example of how influential such risk interactions can be comes from the worst pandemic in the modern era:^21^ the 1918 influenza pandemic. Death estimates for this event are close to 3–5% of global population, but contextualization is needed.^22^ The 1918 pandemic coincided with the end of a global war; the First World War created the ideal conditions for the spread of influenza among a war‐weary population. Moreover, the war itself appears to have been given a low probability by contemporaries as judged by financial markets (Ferguson, 2006), this is despite historians documenting various international crises in years preceding the war. In contrast to the 1918 influenza pandemic, the less heralded global influenza pandemic of 1889–92 had a much lower death toll, with no other coinciding global phenomena. This demonstrates the dangers of extrapolating lessons from 1918 and applying them to modern events without contextualization‐ the pandemic was undeniably exacerbated by the global war—but at the same time, this illustrates the importance of the interaction of risks (Doran et al., 2024).

The interaction of risk has led to new understandings of risks that deviate from conventional risk management, such as the cardiovascular disease epidemic in the latter twentieth century, prominent among these are the study of ‘‘systemic risks’’ (Helbing, 2013; Renn et al., 2022). A now classic definition of systemic risk as a breakdown of an entire system rather than individual components of the system was originally applied in the context of financial systems (Kaufman & Scott, 2003). However, with increasing globalization and interconnectedness, the application has spread to various global risks (Lucas et al., 2018). While systemic risk has not been fully integrated in either PB or GCR framework, it is clearly applicable and some aspects of both risk paradigms (e.g., pandemics from infectious diseases, environmental risks, and risks from technology) are considered in early applications of systemic risk thinking (e.g., OECD, 2003). Although as systemic risks tend to be the interaction of individual risks, they tend not to receive the same level of attention as GCRs or PBs (Renn et al., 2022). Richardson et al. (2023) see PBs as being systemic, but this refers only to the Earth System, on which the PBs are based, and not spillovers to other risks or other systems. However, thinking of risks as interacting stresses the importance of not siloing thinking on risk. Effectively, both PBs and GCRs can be seen as extreme systemic risks as they threaten entire systems and the dynamic in which the risks are realized is either endogenously (through failures within the system) or exogenously (external attacks to the system) (Hochrainer‐Stigler, 2020). Many of the PBs outlined are endogenous to human society, they are a result of the human system, while many GCRs are exogenous there are some that are endogenous. It is the endogenous risk where PBs and GCRs overlap.

A relevant modern example is the Bovine Spongiform Encephalopathy (BSE) outbreak in the United Kingdom in the 1980s and 1990s, which resulted in an EU ban on UK beef exports that was only lifted as late as 2006. The BSE event was influential and explicitly referenced in the early work of systemic risk given it revealed stresses in industrial scale agricultural production (e.g., OECD, 2003; Renn & Klinke, 2004). This event was caused by the declining profitability of beef farming, which resulted in efforts to economize on cattle feed through the use of meat and bone meal (Collee & Bradley, 1997). BSE's human variant, variant Creutzfeld Jacobs Disease caused 178 deaths (Beck et al., 2005; National CJD Research and Surveillance Unit, 2020). Remarkably, the relatively low number of human deaths resulting from BSE's human variant is still unexplained, given that worst case scenarios predicted up to 150,000 fatalities for the United Kingdom alone (Ferguson et al., 2002).^23^ The BSE episode therefore does not fit into the PB framework. It does, however, align with the GCR as it is an example of a new disease derived from the unanticipated consequences of human activity; however, the realized fatality rate does not meet the threshold of a GCR.

One of the potentially most alarming scenarios concerns a situation where catastrophic or gradual climatic events trigger a major pandemic, which, in turn, affects food supplies and the supply of other health relevant goods such as pharmaceuticals. Shortages of key pharmaceuticals have already occurred, and the underlying complex global manufacturing supply chain has shown vulnerability to natural disasters. For example, in 2017, Hurricane Maria destroyed much of the injectable production capacity in the United States, causing shortages of intravenous saline (Slacks et al., 2018). Similar problems now occur due to the Covid‐19 pandemic (Choo & Rajkumar, 2020).

**FIGURE 7:**
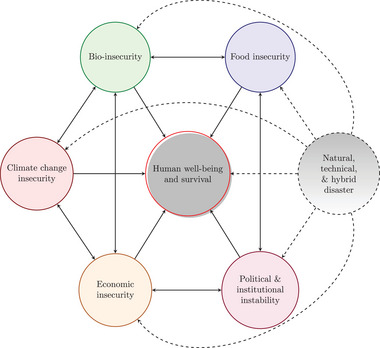
Schema of interlinked risks. *Note*. At the center of the figure is human well‐being and survival that is affected by the various insecurities directly and indirectly from the disaster. The circle representing natural, technical, and hybrid disasters is open, indicating that the disaster comes from elsewhere and is not contained. The thicker arrows represent the direction of the effect from the disaster on the preexisting network of insecurities; the solid line represents a direct effect on human health and survival, while the dashed lines represent indirect effects through their impact on the various insecurities. The relationship between various insecurities is illustrated using double line arrows so as to highlight the two‐way processes in which these relationships are interacted.

It is therefore possible to imagine an outbreak of a pandemic coupled with a lack of key medication due to a breakdown of supply chains. Such a situation could interact with a natural disaster that further exacerbates the death toll through ripple effects, leading to food shortages and widespread regional deprivation.^24^ Potential global worst‐case scenarios can arise where existing networks of insecurities in the areas of politics, economics, climate, food, biological safety, and food supply are aggravated by the occurrence of one or several major natural, technical, or hybrid disasters^25^ (as illustrated in Figure 7). Figure 7, drawing on McMichael (2013), refers to the linkages of global risks and the potential impact of disasters in simplified form, given the key argument about the connectivity of core global risks.^26^ The main implication is that the GCR‐PB is not part of the normal network of insecurities but that it affects each and these, in turn, interact with other insecurities. The dynamics of these interactions are hidden in this figure as these interactions happen over time and are not instantaneous. The question then is how quickly these insecurities respond to changes.

The idea of interlinkages of risks is not new and has been conceptualized in older literatures.^27^ The original PB style report, the 1972 *Limits to Growth*, emphasized dynamic interactions (Meadows et al., 1972). A follow‐up report commissioned by the Carter Administration also alluded to dynamic interaction, noting that:

… These interrelated issues [economic development, population growth, resource depletion, and environmental deterioration] not only can lead to social disruptions, economic instability, and political unrest within individual countries, but also can be a major cause of tension and armed conflict across national borders…Unfortunately, short‐term approaches usually fail to take into account adequately the linkages among sectors and, as a consequence, eventually lead to other, often more serious, problems (Barney, 1980: 4).

Despite the potential for the interaction of risks, Garrett (1994) highlights how the Cold War distracted policymakers from microbial and ecological risk. This was aggravated by a focus on economic growth (production and output), encouraged initially by the Marshall Plan, and later by an assumption that infectious diseases that might occur in the Third World (i.e., political nonaligned), countries could easily be controlled by mass immunization. Crucially, these assumptions tended to focus on single risks. Garrett (1994) notes that an increasing globalization of trade and travel increases the risk of the spread of infectious disease (see also Beltz, 2011). Needless to say, these problems occur in tandem with other adverse environmental effects of globalization, such as increased emissions from trade, habitat destruction, inadvertent spread of invasive species, deforestation, and decreased biodiversity.

This brings in the question of the trade‐off between stability and resilience as found in ecological systems, and where more complex systems can fluctuate more rapidly than less complex ones (Holling, 1973). In this sense, complex societies can be seen as being at greater risk due to increasing interdependence with more exposure to risk as the level of complexity increases. Take, for example, the supply chain shocks during Covid where complex “just in time” models, already identified as vulnerable to shocks (Centeno et al., 2015), were the nodes through which Covid shock permeated across the global economic system. Scheffer et al. (2023) argue that increasing complexity led to reduced resilience in premodern societies and was a factor in their decline. While modern complex societies may suffer from similar ills (increased inequality, environmental degradation, and population increase), they are also better at predicting risks and therefore can be more resilient than past societies.

## 6 FOOD SECURITY AND PANDEMICS

**FIGURE 8:**
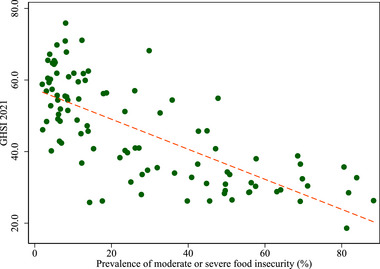
Scatterplot global health security and food insecurity. *Note*. There are 99 countries with data available for both GHSI and food insecurity. Each green dot represents a country. The dashed line indicates a fitted line.

Here, we illustrate the overlap between PBs & GCRs using the example of food security, which refers to stable access, availability, and utilization of safe and nutritious food.^28^ The health effects of food insecurity have brought it within the realm of public health (Knowles et al., 2018), and it is seen ‘‘an underrecognized social determinant of health’’ (Murthy, 2016). For example, see Figure 8 that highlights the negative correlation between a country's global health security index ranking and its food insecurity.

As domesticated food sources, both plant and livestock, have evolved in prevailing climatic conditions, and climate change and extreme weather events can undermine food security (McMichael & Butler, 2011). The Food and Agriculture Organization (FAO) of the United Nations reports annually on food insecurity, and it is clear that agriculture is much exposed to the vagaries of climate, for example, as manifested in the increased drought conditions of 2021 (Langenbrunner, 2021). Therefore, food security is threatened directly by climate change (IPCC, 2018; IPCC, 2022), in part because increased agricultural activity impinges on other global environmental parameters via land use change (deforestation) and biodiversity loss (Garrett, 1994; de Castro Solar et al., 2016), while also being an important contributor to greenhouse gas emissions in the form of methane from livestock that has accelerated in recent years (Cheng & Redfern, 2022). Recent meta‐analyses of climate change damages suggest that estimates of future damages can be four times greater than is currently assumed (Howard & Sterner, 2017). Moreover, recent estimates predict weather disruptions will significantly impact on economic activity, which directly affects agriculture and can have indirect impacts on regions less dependent on agriculture as a source of income (de Winne & Peersman, 2021).

The GCR literature suggests there could be feedback between GCRs and food security (Helfand, 2013). Huff et al. (2015) explored the potential impact of a pandemic on the U.S. food system, finding that the food system was not resilient to an extreme pandemic event that led to worker absenteeism of between 20% and 40%. Evidence from the Covid‐19 pandemic appears to support this finding, as world hunger worsened with between 9% and 10% of the world's population experiencing hunger in 2021 (FAO, 2021b); this increase in food insecurity was not necessarily due to morbidity from the pandemic itself rather it was a result of the fallout from policy responses (i.e., nonpharmaceutical interventions).

Animals provide a sizeable share of the food supply of the planet. Yet, animal populations can experience catastrophic levels of disease that can affect supply chains and human health in a number of direct and indirect ways (Vilanova et al., 2019). For example, African swine fever (ASF) is a highly infectious viral disease that mainly affects domesticated pigs and is spread from wild boars (OIE, 2019). It was first discovered in Africa in the early 1900s and spread beyond Africa in the early 2000s to the trans‐Caucasus region of Russia and Eastern Europe (see Sánchez‐Cordón et al., 2018) and on to China, the world's largest pork producer (Shao et al., 2018).

This relates to the question of interactions in relation to food security and health. China experienced a devastating epidemic of ASF from 2018–2021 that decimated an estimated 40% of the country's swine population (Gale et al., 2023; Normile, 2019; Zhou, 2019; Zhou et al., 2019). Farmers were forced to switch to poultry and canines as a protein source, causing increased meat prices, and leaving more than 8% of the Chinese population malnourished (Beck & Tobin, 2020). A study of the ASF outbreak in China indicates how cases were located in provinces neighboring Hubei—the epicenter of the Covid‐19 pandemic (Wang et al., 2018)—and ASF continued to be a lingering problem in these areas (Shike, 2020). Estimated impacts of ASF include loses of 0.78% to Chinese GDP and greater damage to pig herds than those reported in official estimates (You et al., 2021). Food insecurity can create vulnerabilities to climatic events (Hu et al., 2017) including natural disasters in the broadest sense, with the lurking danger that agricultural mismanagement will create hazards to human health. A related worry with Chinese pork production is the abuse of antibiotics as a growth promoter leading to antibiotic resistance (see Yang et al., 2019). The ASF episode highlights the importance of holistic approaches to health, such as One Health that focuses on both human, veterinary, and environmental health (Chien, 2013; WHO, 2017); although the novelty of the term hides the long history of medical veterinary interactions (Woods & Bresalier, 2014).^29^

Coronaviruses are a family of viruses that mutate and infect other species—‘‘cross‐species transmission’’—and are quite common. For example, variants of coronavirus were identified as infecting and wasting piglets in the 1980s and again in the early 2000s (Quiroga et al., 2008; Turgeon et al., 1980). Livestocks are a potential target and also a source of communicable disease. The event of a health shock to livestock may force people to scavenge for food from untried meat sources, risking exposure to new viruses. Evidence that wild food is frequently an alternative to farmed meat comes from bushmeat consumption and trade, where poverty is a strong driver in demand (e.g., Fischer et al., 2014; Lindsey et al., 2013). Bushmeat has been linked to epidemics, such as Ebola, which resulted in bushmeat bans during epidemics, whereby belated bans may have had the unintended consequence of further reducing public confidence in public health responses (Bonwitt et al., 2018).

There is no direct evidence that climate change has affected the transmission of coronaviruses, but it has had indirect effects on the movement of animals, which creates opportunities for pathogens to cross species (Rohr et al., 2019). Deforestation is also an indirect transmission path, as land use change can bring wild animals into contact with domesticated animals (Afelt et al., 2018). Such dynamics increase the risk of transmission of zoonotic diseases, diseases caused by viruses, bacteria, or fungi that spread between animals and people. Examples of zoonotic diseases are the 2009 H1N1 influenza pandemic that originated in swine and infected both pigs and humans (Scotch et al., 2011) and also smaller events such as the Chapare Hemorrhagic fever episode in Bolivia in 2019 (Mafayle et al., 2022) or the 2022 Ebola outbreak in Uganda (Kozlov, 2022) that coincides with acute food insecurity pressure. The WHO's 2015 list of top emerging diseases was all zoonotic in nature (Morens & Fauci, 2020; WHO, 2015) and early genome analysis of Covid‐19 points to zoonotic origins of the pandemic, as viruses in bats and Malaysian pangolin had a 99% similarity to SARS‐CoV‐2 (Andersen et al., 2020; Hassanin, 2021). Although the exact mechanism how Covid crossed into humans is still debated as per letters in *Science* and the *Lancet* (Bloom et al., 2021; Calisher et al., 2021; van Helden et al., 2021), most recent work indicates zoonotic origins (Worobey 2021).^30^ However, the intense politicization (including the rush to discredit the lab leak thesis among some) means that it is difficult to determine if Covid‐19 was zoonotic (GCR‐PB) or man‐made (GCR).^31^ In any case, there is mounting evidence that climate change is increasing the spread of zoonotic disease (Carlson et al., 2022).

Researchers have long highlighted links between climate change (viewed often as PB) and public health, although there has been a noted change in emphasis and tone of this research over the past 40 years (Butler, 2018).^32^ Accordingly, climate change increases the risks of vector‐borne disease incidence (McMichael et al., 2003, Campbell‐Lendrum et al., 2015; ICS 2017), with evidence including a worsening of the global malaria burden alongside other diseases such as dengue fever^33^ (McMichael, 2017).^34^ This emphasizes environmental influence, or what Frohlich et al. (2001) label context, on public health outcomes. One problematic aspect of viewing these relationships as manifestations of PBs is that, at least for some, these PBs then cease to be considered a global threat. This is exemplified by Edwin Kilbourne,^35^ who optimistically suggested: *pandemics, if they occur, will be primarily from respiratory tract pathogens capable of airborne spread. They can be quickly blunted by vaccines, if administrative problems associated with their production, distribution, and administration are promptly addressed and adequately funded* (2008, p. 303). Similarly, in a recent reappraisal of the Covid‐19 response, Morens et al. (2020) concluded that humanity had entered a ‘‘pandemic era’’ but were buoyed by the diagnostic testing apparatus and the quick roll out of vaccines as indicating readiness to address future pandemics but that this scientific advancement was ‘‘*dramatically insufficient to prevent and control their emergences in the first place*.’’ This techno‐optimism is somewhat at odds with the wider scientific community that has been concerned about pandemics as well as increasing microbial resistance to antibiotics (e.g., Garrett, 1994; Institute of Medicine, 1992; Murray et al., 2022). An associated issue with seeing vaccines as a pandemic control measure is that vaccines may not be developed quickly, as in the case of HIV/AIDs, or the absence of good vaccines for other diseases prone to flareups, such as TB and dengue.

Decreased certainty about our ability to ‘‘quickly blunt’’ disease risk has made technological optimism problematic and should throw doubt on drawing lines around PBs that distinguish these from more immediate GCRs. Simply put, is there not a strong possibility that any pandemic, once in full swing, could, in fact, result in a global catastrophe? And is it not dangerously naïve to assume that ‘‘administrative problems’’ associated with vaccine ‘‘production, distribution, and administration’’ would be easy to resolve? For example, an unanticipated issue with the Covid vaccines is vaccine hesitancy. This should have unsurprising given trends in antivaccination movements but more directly because of the association of the Guillain–Barré syndrome with the 1976 swine flu vaccine program in the United States (Evans et al., 2010); Edwin Kilbourne was principal advisor to the U.S. president on that particular vaccination program and wrote an Op‐Ed in the *New York Times* warning of a worldwide flu pandemic (Martin, 2011).

## 7 MANAGING WEALTH AND MITIGATING RISK

**FIGURE 9:**
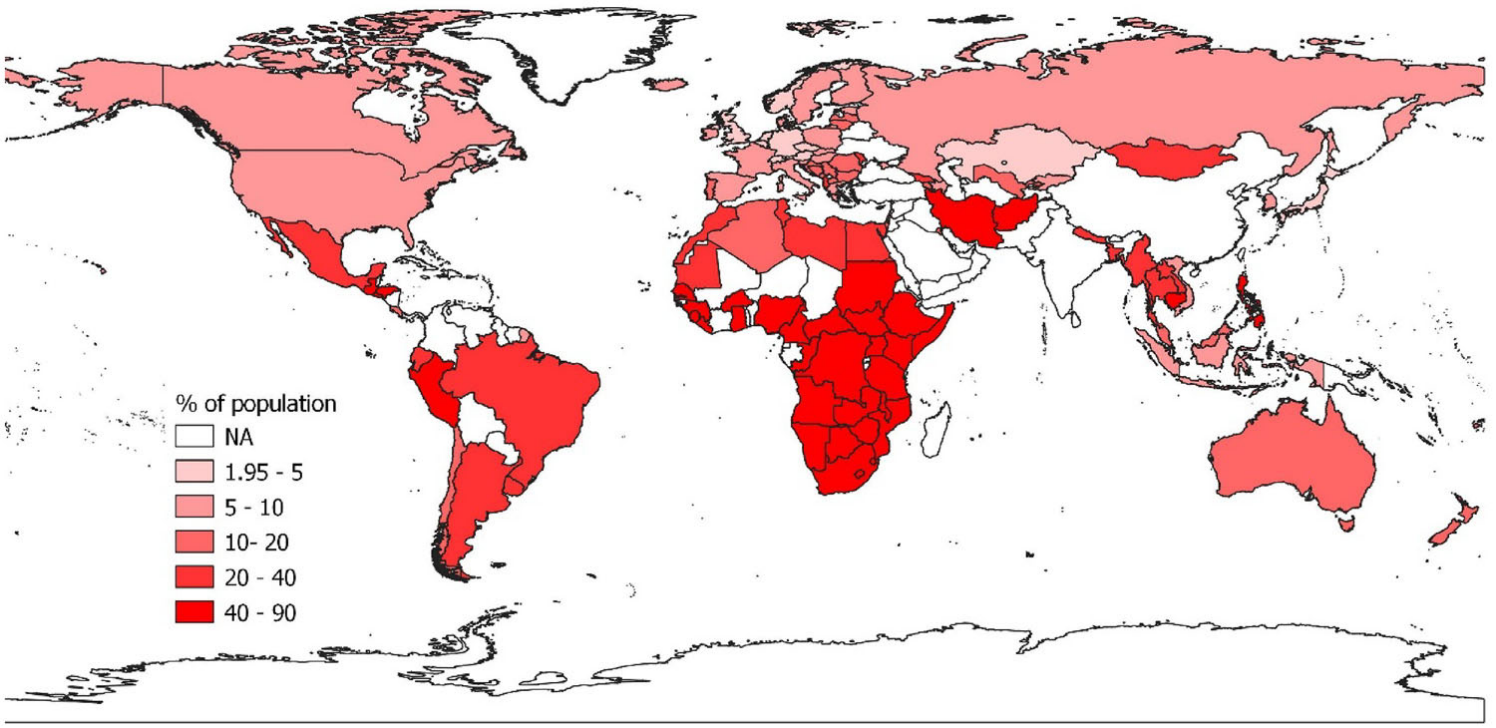
Prevalence of moderate or severe food insecurity (%) of population. *Source*: Data from Food and Agricultural Organisation (FAO) (2021a). Mapped using QGIS.

The planet has been warming due to human activity, which has been documented by the Intergovernmental Panel on Climate Change (IPCC) (2021, 2022). The number of natural disasters rose dramatically since the 1960s (GAR 2023; OECD, 2003) with increasing evidence that climate change is driving the intensity and frequency of natural disasters (Coronese et al., 2019) and this increased risk of natural disasters (Figure 8) will have an impact on food insecurity, which already shows signs of strain (see Figure 9).^36^ For example, Cooper et al. (2019) find that extreme drought conditions were associated with worse child nutrition in developing countries and the results were evident in regions deemed to food insecure. Similarly, Palmer et al. (2023) highlight how disruptions to rainfall in Somalia in 2016/17 led to acute food shortages and malnutrition. In 2020, global economic losses due to natural disasters were among the worst on record (MunichRe, 2021). Country‐specific estimates also highlight the increasing costliness of weather and climate disasters, for example, the US National Oceanic and Atmospheric Administration (NOAA) (2023) shows that there were 18 separate weather and climate disasters in the U.S. costing at least $1 billion in 2022.^37^ While many of these natural disasters would have occurred in the absence of climate change, Newman and Noy (2023) estimate that at least 53% of total damages can be attributed directly to climate change and that much of this damage is due to loss of life.

The prevalence of moderate or severe food insecurity is shown in Figure 9. 38 FAO (2023) reports a 4‐percentage point increase in moderate or severely food insecurity globally following the Covid‐19 pandemic; however, there was significant geographic variation.^39^

How can we manage and mitigate these risks? Dasgupta (2021) places emphasis on insuring against risk. But one of the biggest challenges here is the global nature of risk and the associated moral hazard from individual countries not contributing to the global public good. This highlights the importance of maintaining the integrity of natural capital as means for maintaining the welfare of humanity (Guerry et al., 2015).

The *Dasgupta Review* (2021, p. 5) argues that “in order to judge whether the path of economic development we choose to follow is sustainable, nations need to adopt a system of economic accounts that records an inclusive measure of their wealth.” Dasgupta draws on the economic literature regarding sustainable development, which, in turn, was inspired by the Meadows et al. (1972) *Limits to Growth* report. The economics of sustainable development focus on the means to achieve future well‐being (Hanley et al., 2015). The theory focuses on *IW*, in particular, the change in *IW*, as a predictor of future well‐being (Dasgupta, 2001). The intuition being that each generation is a custodian of *IW*, and for future generations to derive the same level of well‐being, this *IW* must be maintained or augmented. *IW* is seen as central to understanding the nature‐society interactions within the wider sustainability literature (Clark & Harley, 2020). *IW* includes all assets from which people obtain well‐being, either directly or indirectly (Dasgupta, 2001). Thus, *IW* measures the value of all forms of capital (produced, natural, and human) in a country. Changes in *IW* per capita, whether positive or negative, are indicators of sustainable or sustainable/unsustainable development (Hanley et al., 2015). The World Bank (2021) now follows this view and suggests that focusing on a change in *IW* per capita could help manage risk and uncertainty. *IW* can be monitored at different scales (global, regional, national, and subnational) and this can help target mitigation efforts.^40^

The distinction between GDP, the traditional measure of economic activity, and *IW* is perhaps illustrated more clearly by comparing their accounting identities, as shown in Equations (1) and (2):

(1) $$GDP_{t}=C_{t}+I_{t}+G_{t}+NX_{t},$$

(2) $$IW_{t}=\sum_{i}\left(p_{it}K_{it}\right)+\sum_{j}\left(h_{jt}H_{jt}\right)+\sum_{k}\left(p_{kt}S_{kt}\right)+\sum_{m}\left(p_{mt}Z_{mt}\right),$$

where GDP is the sum of consumption (C), investment (I), government consumption (G), and net exports (NX), while *IW* is composed of the value (price *x* quantity) of different forms of capital; manufactured (K), human (H), natural (S), and knowledge (Z) (Dasgupta, 2001, eq 9.1). The relationship between the two concepts is that *IW* is seen as the foundation of future income (GDP) (see figure 4), which effectively sees a country's income as a return on its assets (*IW*).^41^ The change of *IW* makes this clearer as the change in *IW* is effectively investment (I in Equation (1)) although as made clear in Equation (2), investment would be thought of more broadly in *IW*.

**FIGURE 10:**
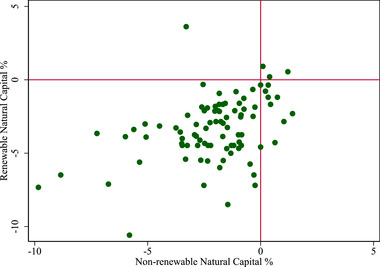
Growth in renewable and nonrenewable natural capital per capita.

*IW* can also be analyzed in terms of its subcomponents with specific attention given to natural capital to identify trends in natural capital. Within natural capital, this is classified as renewable (agricultural land, fisheries, forestry resources) and nonrenewable natural capital (fossil fuels and minerals), a scatterplot of the growth in both renewable and nonrenewable natural capital per capita is presented in Figure 10. The growth of natural capital presents a different narrative to that of IW as a whole (cf. Figure 3) and therefore could be used as a distinct indicator in its own right. The present system of accounting values all forms of capital and aggregates them in an unweighted index. In principle, weights could also be applied to the indices to focus on the elements of natural capital that are deemed critical. For example, within ecology, keystone species have a disproportionate effect on an ecosystem relative to their abundance (Denno & Lewis, 2009; Power et al., 1996) and there have been various keystone species identified in different ecosystems (Shukla et al., 2023). Weighting keystone species within a natural capital framework would thus be in line with existing studies that propose weights to measure keystone communities, although with the caveat regarding the choice of weight (Mouquet et al., 2013). Thus, there is scope for further research into these metrics and to develop more complete measures (e.g., see Fenichel, 2024).

**FIGURE A1:**
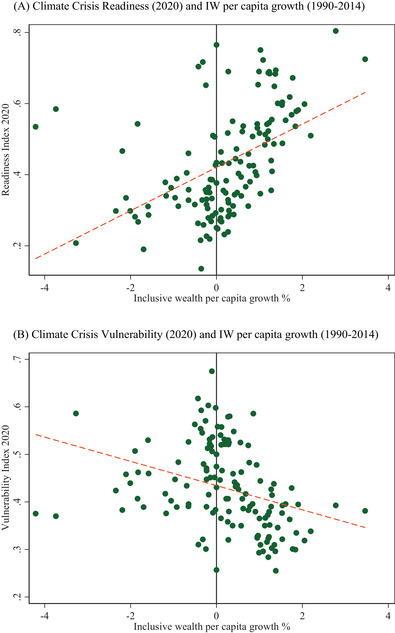
Scatterplot of climate risk and growth in IW per capita. *Note*. Each green dot represents a country, and there are 137 countries included. The dashed line indicates a fitted line. Sources: UNEP (2018) & ND‐GAIN (2022).

**FIGURE A2:**
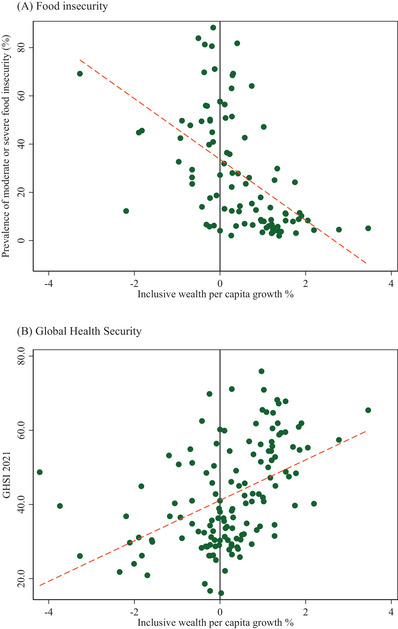
Scatterplots of food‐health security and growth in IW per capita. *Note*. Food security is measured in 2018–2020. There are 99 countries with data available for both food insecurity and IW per capita. Each green dot represents a country. The dashed line indicates a fitted line. Global Health Security Index in 2021. Each dot represents a country. There are 137 countries with data on GHSI and IW. The dashed line represents a fitted line. Sources: FAO (2021a), Global Health Security Index (GHSI) (2021), & UNEP (2018).

The economic theory on which *IW* is based stresses that changes in *IW* are an indicator of future sustainability, defined as nondeclining well‐being (Hanley et al., 2015). Therefore, in crude terms, changes in IW should be correlated with metrics of interest to future well‐being. In essence, then *IW* can be used to monitor risks endogenous to our economic systems. Using estimates of *IW* per capita growth from United Nations Environment Programme (UNEP) (2018) illustrates how maintaining or increasing *IW* per capita is a useful indicator of future climate readiness and vulnerability (Figure A1),^42^ food security, and health security (Figure A2). Therefore, one approach to mitigating risks from GCR‐PBs is to monitor *IW* as an indicator of possible endogenous risks.

## 8 CONCLUSION

The Covid‐19 pandemic has raised awareness of low‐probability but high‐impact events. Estimates of the economic cost of Covid‐19 in the United States alone put the impact, both direct and indirect, at $16 trillion, which equates to 90% of annual U.S. GDP, an amount greater than U.S. expenditure on wars in the 21st century and equivalent to 50 years of climate change damages (Cutler & Summers, 2020). Scaled up at a global level, the immediate economic cost of Covid‐19 was enormous and easily surpasses the $10 trillion threshold for a GCR laid out by Bostrom and Ćirković (2008).

Such high‐impact events have previously been conceptualized using either PB or GCR frameworks. We argued for a more holistic framework that encompasses a multitude of risk and draws on expertise from both frames. Going back to Figure 1 where we highlighted the two isolated approaches to existential risk. Our proposal is to situate the PB framework *within* the GCR frame (Figure 2), effectively replacing overlapping risks such as climate change and offering a broader understanding of risks. Ultimately, we call for greater interaction and engagement between both frameworks in order to have a deeper understanding of existential risks.

We highlight the difficulty of categorizing risk given the interactions among different risk types (see Table 1). Our focus was on public health aspects of the overlap between PBs and GCRs. Verner et al. (2016) argue that research on the health risks of climate change is underdeveloped. Interactions between health systems and climatic shocks can leave us vulnerable. Some health impacts can be mitigated proactively (Wu et al., 2016) and scientific knowledge advances might occur (Kingsland, 2020). But this requires timely interventions and capable national and multinational institutional frameworks.

As shown by Covid‐19, new diseases create immediate challenges for which we need to be prepared while we await vaccination to diminish a disease threat; indeed, for some pandemic diseases (HIV/AIDs), there are still not vaccines developed. Increasing microbial resistance to antibiotics aside, it has now become painfully obvious that humanity has not conquered the threat of infectious diseases. This recognition of an increased threat of disease means additional resources that will need to be directed to this, which will intensify pressures within the existing network of global risks.

A key message for policymakers, activists, and researchers is to think broadly about how we define and classify risks. In the specific case of pandemics, this requires us to think how to address the causes of a pandemic (including degradation of our natural capital) while treating its symptoms. In our view, one necessary step in acquiring the requisite broad perspective on global risk will be to question the idea that we can accurately define PBs and separate these from GCRs in a meaningful way, against the background of an inclusive meaningful concept of wealth (*IW*). Removing artificial boundaries to risk typology would be one first step to have a more holistic understanding of potential existential risks.

We have highlighted how changing focus from monitoring a country's national income (i.e., GDP) to monitoring the *IW* of a nation could help mitigate GCR‐PB risks that may be endogenous to economic systems. The benefit of this approach is that it places greater emphasis on natural resources, such as ecosystem services and biodiversity, which tend to be undervalued in traditional economic decision making. In doing this, we effectively endorse the recommendations of the *Dasgupta Review* (2021) and encourage a greater use of these initiatives beyond narrow academic circles to policymakers and international organizations. Both the UNEP and World Bank currently provide estimates of *IW* but policymakers need to be made aware of the wider benefits and applications of these estimates.

Lastly, many of the models of PB and GCR have given a limited role to human agency, the models are purely deterministic, and humans are effectively like animals (Hochrainer‐Stigler et al., 2019). Some of these have led to overly optimistic solutions such as that outbreaks of infections will inevitably result in the production of a cure or vaccine or extremely pessimistic views that systems will collapse. Social systems are adaptive and composed of heterogenous agents thus giving greater role for human responsiveness will be key to developing realistic models of GCR‐PBs. Following Hochrainer‐Stigler et al. (2019), continuous monitoring of *IW* can help mitigate potential risks that arise endogenously.

## Data Availability

Data are publicly available and researchers will share data used to generate figures.

## Footnotes

1 For example, see Economist (2020), Ferguson (2021), Tooze (2022), and Achenbach (2022). There is a longer tradition of doomsday prognostication that predates current concerns (e.g., Jha, 2011).

2 In our, we focus on risks as specific hazards rather than as a probabilistic concept.

3 There are different definitions within the field. For example, Karger et al. (2023) refer to 10% of population while also noting that there is no agreed definition (p. 22). However, definitions have caused debate (e.g., Beard et al., 2020a, 2020b; Baum, 2020). Torres (2023) in his survey of various definitions argues for an agreed definition for communicating with nonexperts.

4 Oliu‐Barton et al. (2021) compared GDP growth and Covid‐19 outcomes for OECD countries that implemented elimination strategies (Australia, Iceland, Japan, South Korea, New Zealand) with those that implemented mitigation strategies. They found that countries with more stringent controls had slightly higher economic growth; however, the data of economic growth were real‐time estimates of economic output based on Google trends (Woloszko, 2020). Revisiting the analysis using data on growth of real GDP per capita shows that elimination countries did perform slightly better in 2020 at −3.56% growth compared with −4.65% for nonelimination countries, but in 2021, elimination countries rebounded slower than nonelimination countries (3.41% vs. 6.70%) supporting Stiglitz's observation. In any case, countries that choose the elimination strategy might have been economically stronger than the other group, so policy selection might have biased by the nature of each sample group.

5 For example, see the extensive treatment by Dasgupta (2001) and Weitzman (2003).

6 Beck and Grande (2010) call for a cosmopolitan turn that many transnational organisations now seem to pursue. *IW* measures are driven by transnational organisations rather than nation states.

7 Jones (2023) adopts a similar to approach weighing up economic growth and AI risk (a GCR) uses a utility/well‐being approach. There is also an earlier literature that emphases on the multitude of catastrophic risks and how to think broadly about how to respond (Martin & Pindyck, 2015).

8 The ‘‘2oC guardrail’’ target first proposed by William Nordhaus is a risk‐averse boundary not derived from modeling but from considering the variation in long‐term climate in the 1970s and concluding that 2 was outside the range of human experience (Nordhaus, 2018).

9 This also touches on arguments for and against ending the Holocene and declaring an Anthropocene that is still widely debated (e.g., see Monastersky, 2015; Subramamian, 2019). While a proposal for a formal recognition of the Anthropocene as a new epoch in the Earth's geological timeline was formally rejected in 2024 by a subcommission of the International Commission on Stratigraphy, Anthropocene still exists as a cultural concept (Witze, 2024).

10 Fragile states refer to an index aiming to assess a country's risk of conflict or implosion. It is a composite index based on five indices with each index composed on subindices. The scores of countries are ranked out of total of 10 and all composite indices are added to give a final score. The indices are: Cohesion: C1: Security Apparatus; C2: Factionalized Elites; C3: Group Grievance. Economic: E1: Economy; E2: Economic Inequality; E3: Human Flight and Brain Drain. Political: P1: State Legitimacy; P2: Public Services; P3: Human Rights. Social: S1: Demographic Pressures; S2: Refugees and IDPs. And Cross cutting: X1: External Intervention. https://fragilestatesindex.org/global‐data/.

11 The Global Challenges Foundation (2021) publishes an annual assessment of catastrophic risk.

12 Baum et al. (2019) include an update with several additional scenarios included to the schema.

13 Here, we make a distinction between humanity and social subgroups such as those identified in Diamond (2005). In the latter, Diamond focuses on various geographically isolated societies in premodern period.

14 The mortality rates from the Justinianic Plague range from 25–50% that make it comparable to the Black Death (Alfani, 2024). However, there has been recent debate downplaying the impact of the pandemic—see Sarris (2022).

15 Vectors are living animals that transmit infectious pathogens from animals to humans or between humans (WHO, 2020).

16 For example, there were several cases of autochthonous cases of dengue transmission in France in 2022 and in Italy in 2023 (Cochet et al., 2022; EpiCentro, 2023).

17 Bats, one of the main concerns for zoonotic spillovers, are threatened because of habitat loss as well as hunting (Tanalgo et al., 2023). However, we are purposefully not discussing complex interactions such as the possibility of further habitat destruction causing the extinction of bats and thereby reducing the threat of possibility of zoonotic spillovers from bats. Because such an extinction scenario would likely lead to zoonotic spillovers as the animals themselves attempt to adapt.

18 Similarly, but somewhat distinct, King and Jones (2021) refer to ‘‘risk multipliers’’ that ‘‘exacerbate existing trends and feedbacks’’ and they see climate change as the main example of a risk multiplier that ‘‘interfaces’’ with human and natural systems.

19 Earth system science on which PBs are based is an application of system dynamics and thus focuses on interactions within the Earth system (Steffen et al., 2020).

20 Nonlinearity (results out of proportion to the stimulus), open environment (interact with its environment), self‐organisation (e.g., multiple interactions and recursive feedback), emergence (collective is more complex than the individual due to interactions), patterns of interaction (unpredictable interactions), adaptation and evolution, and coevolution (Sturmberg & Martin, 2013).

21 Estimated mortality rates (c. 20–40%) were higher during the Plague of Justinian, the Black Death, and the introduction of smallpox to the Americas. Doran et al. (2024) argue that these are unrepresentative of future pandemics owing to the fact that the world has become more global and also because of improvements in medical knowledge about disease transmission and public health management practices in the modern era.

22 It must be noted that the literature on pandemics is dominated by the 1918‐influenza pandemic (Doran et al., 2024) and that there were several other influenza pandemics in the subsequent century that have received much less attention.

23 This figure reflected the uncertainty surrounding the extent of exposure of the wider livestock sector with worst case scenario estimates of mortality of sheep exposure at 150,000 (Ferguson et al., 2002).

24 These are scenarios that development practitioners already viewed with the greatest of worry in the early part of the Covid‐19 pandemic (e.g., Ngumbi, 2020).

25 Here, a hybrid refers to a natural disaster interacts with another risk, for example, Fukushima Japan where an earthquake and Tsunami disabled the cooling system of a nuclear reactor (Beck, 2019).

26 This is also similar to the visualisation of a systemic risk in Hochrainer‐Stigler (2020, figure 1.4) that sees a system comprised as a network that is composed of individual risks and dependencies. The nodes are the individual risks and the connections highlight the dependencies between the risks.

27 Similar arguments were developed in the 1970s in the writing of French and German environmentalists. See Beck and Kewell (2014) for a discussion of the history of risk.

28 Concerns about this issue are long standing (see Sachs, 2015; Stern, 2006).

29 One Health is conceptually similar but distinct from “planetary health” (see Lerner & Berg, 2017).

30 This is a controversial topic with incomplete information. But that the latest information at the time of manuscript writing (March 2023) did favor animal origins (Cohen, 2023). An article published in September 2024 supported the animal hypothesis (Crits4‐Christoph et al., 2024), but this has been disputed (e.g., see Woolhouse, 2024).

31 For example, the continuing Congressional investigations (US, 2023) into the initial publication that aimed to quash the idea of a lab‐leak (GCR) (Anderson et al., 2020) highlight conflicts of interest of researchers who conduct research that may lead to possible lab‐leaks (GCRs) (van der Merwe, 2023), this is countered by the efficacy of such research as part of a one health strategy for pandemic preparation that sees zoonotic origins of pandemics (GCR‐PB) (Daszak, 2023).

32 Institute of Medicine (1992, p. 76) was warning about effects of global warming on disease transmission, which was frustrated by ‘‘the apparent lack of interest in global warming as a possible contributor to public health crises on the part of funding agencies and environmental groups.’’

33 For example, there were several cases of autochthonous cases of dengue transmission in France in 2022 (Cochet et al., 2022).

34 Malaria is climate sensitive and there have been various studies suggesting an increased risk of malaria spread with climate change, for example (Caminade et al., 2014). In practice, the evidence is mixed as the risk of spread is being countered by countered by the increasing number of countries that have achieved an elimination of malaria and 23 countries are certified as having eliminated malaria from 2000 to 2020 (WHO, 2021c).

35 Edwin Kilbourne (1920–2011) was a preeminent influenza virologist of the twentieth century; see his obituary by Anthony Fauci, in the *Journal of infectious Disease* (Taubenberg et al., 2011).

36 WMO (2021, table 1) illustrates the differences between disasters in terms of mortality and economic losses. Many of the top 10 disasters either meet the Bostrom and Ćirković (2008) GCR threshold in terms of mortality (the lowest is 28,700 deaths and the highest was 300,000) or damage ($25.17 billion to $163.61 billion).

37 See also 2022 U.S. billion‐dollar weather and climate disasters in historical context: https://www.climate.gov/news‐features/blogs/beyond‐data/2022‐us‐billion‐dollar‐weather‐and‐climate‐disasters‐historical

38 A household is classified as moderately or severely food insecure when at least one adult in the household has reported to have been exposed, at times during the year, to low‐quality diets and might have been forced to also reduce the quantity of food they would normally eat because of a lack of money or other resources. The FAO data use a food insecurity experience scale. Moderate implies ‘‘insufficient money or resources for a healthy diet; uncertainty about the ability to obtain food; probably skipped meals or run out of food occasionally.’’ For severe food insecurity, this implies ‘‘a person has run out of food; gone an entire day without eating at times during the year’’ (https://www.fao.org/in‐action/voices‐of‐the‐hungry/fies/en/)

39 Latin America and the Caribbean saw the largest increase, with a 6‐percentage point increase between 2019 and 2022 (31.5% in 2019). Africa, the region with the highest reported food insecurity pre‐Covid (52.3% in 2019) saw an increase of 4 percentage points between 2019 and 2022 compared to a 3‐percentage point increase in Asia (21.2% in 2019).

40 See McLaughlin et al. (2024) for a critical appraisal of the UNEP and World Bank approaches of measuring wealth.

41 This comes from how economists perceive income to be derived from a production function based on capital (e.g., Solow, 1956, 1974).

42 Readiness is defined as ‘‘Readiness to make effective use of investments for adaptation actions thanks to a safe and efficient business environment’’ and vulnerability is defined as ‘‘propensity or predisposition of human societies to be negatively impacted by climate hazards’’ (Chen et al., 2015).

## 1

**TABLE A1:** Classification of planetary boundaries.

Earth‐system process	Parameters	Planetary boundary	“Safe”	Upper end	Current level
Climate change:	Atmospheric CO_2_ (parts per million by volume)	350 ppm	280 ppm	450 ppm	417 ppm
	Change in radiative forcing (watts per meter squared)	+ 1.0 Wm^−2^	0 Wm^−2^	+ 1.5 Wm^−2^	+ 2.91 Wm^−2^
Rate of biodiversity loss	Genetic Diversity (E/MSY)	< 10 E/MSY	1 E/MSY	100 E/MSY	>100 E/MSY
	Functional integrity: measured as energy available to ecosystems	HANPP < 10%	1.9%	20% HANPP	30% HANPP
Stratospheric ozone depletion	Stratospheric O_3_	∼ 276 DU	290 DU	261 DU	284.6 DU
Ocean acidification	Carbonate ion concentration, average global surface ocean saturation state with respect to aragonite	>80% Ω_arag_ Of mean preindustrial level	3.44 Ω_arag_	2.75 Ω_arag_	2.8 Ω_arag_
Biogeochemical flows: Phosphorus and Nitrogen cycles	Phosphorus cycle: Quantity of P flowing into the oceans (millions of tons per year); regional: P flow from fertilizers into erodible soils	Global 11 Tg of P year ^−1^; regional 6.2 Tf of P year^−1^	0 Tg of P year ^−1^	Global 100 Tg of P year ^−1^; Regional 11.2 Tg of P year ^−1^	Global 22.6 Tg of P year ^−1^; Regional 17.5 Tg of P year ^−1^
	Nitrogen cycle: Amount of N2 removed from the atmosphere for human use	62 Tg of P year ^−1^	0 Tg of N year ^−1^	82 Tg of P year ^−1^	190 Tg of P year ^−1^
Change in land use	Global: Percentage of global land cover converted to cropland; biome: area of forested land as a percentage of potential forest (% area remaining)	Global 75%; biomes: tropical 85%; temperate, 50%; boreal 85%	100%	Global: 54%; biomes: tropical 60%; temperate, 30%; boreal 60%	Global: 60%; biomes: tropical: Americas 83.9%; Africa 54.3%; Asia 37.5%; temperate: Americans 51.2%; Europe, 34.2%; Asia 37.9%; boreal: Americas 56.6%; Eurasia 70.3%
Global freshwater use	Blue water: human induced disturbance of blue water flow	10.2%	9.4%	50%	18.2%
	Green water: human induced disturbance of water available to plants	11.1%	9.8%	50%	15.8%
Atmospheric aerosol loading	Overall particulate concentration in the atmosphere, on a regional basis	0.1	0.003	0.25	0.076
Chemical pollution	For example, amount emitted to, or concentration of persistent organic pollutants, plastics, endocrine disrupters, heavy metals, and nuclear waste in the global environment, or the effects on ecosystem and functioning of Earth system thereof	0	0	NA	Transgressed

*Sources*: Richardson et al. (2023, Table 1).

*Note*: There are various abbreviations in the table: PPM, parts per million; W m^−2^, watts per meter squared; E/MSY, extinction/million species years; HANPP, human appropriation of net primary product; Ω^arag^, seafloor omega aragonite.
